# Immunological Backbone of Uveal Melanoma: Is There a Rationale for Immunotherapy?

**DOI:** 10.3390/cancers11081055

**Published:** 2019-07-26

**Authors:** Ernesto Rossi, Giovanni Schinzari, Ilaria Grazia Zizzari, Brigida Anna Maiorano, Monica Maria Pagliara, Maria Grazia Sammarco, Vincenzo Fiorentino, Gianluigi Petrone, Alessandra Cassano, Guido Rindi, Emilio Bria, Maria Antonietta Blasi, Marianna Nuti, Giampaolo Tortora

**Affiliations:** 1 Medical Oncology, Fondazione Policlinico Universitario, A. Gemelli IRCCS, 00168 Rome, Italy; 2 Medical Oncology, Università Cattolica del Sacro Cuore, 00168 Rome, Italy; 3 Laboratory of Tumor Immunology and Cell Therapy, Department of Experimental Medicine, Policlinico Umberto I, “Sapienza” University, 00162 Rome, Italy; 4 Ophtalmology, Fondazione Policlinico Universitario, A. Gemelli IRCCS, 00168 Rome, Italy; 5 Pathology, Fondazione Policlinico Universitario, A. Gemelli IRCCS, 00168 Rome, Italy

**Keywords:** immunotherapy, uveal melanoma, checkpoint inhibitors, PD-L1, nivolumab, pembrolizumab, ipilimumab, IDO

## Abstract

No standard treatment has been established for metastatic uveal melanoma (mUM). Immunotherapy is commonly used for this disease even though UM has not been included in phase III clinical trials with checkpoint inhibitors. Unfortunately, only a minority of patients obtain a clinical benefit with immunotherapy. The immunological features of mUM were reviewed in order to understand if immunotherapy could still play a role for this disease.

## 1. Introduction

Standard treatments for metastatic uveal melanoma (mUM) have not been defined yet. Several treatments have been employed with poor results [1,2,3,4,5,6,7,8,9,10,11,12,13,14,15,16,17,18,19,20,21,22,23,24,25,26,27,28,29]. UM was not included in phase III clinical trials with immunotherapy for melanoma, due to the specific biological behavior and the different clinical outcome [3,4,5,6,7,8,30,31,32,33]. Nevertheless, immune checkpoint inhibitors (ICIs) are commonly used for the treatment of metastatic UM [3,4,5,6,7,8].

The available reports with ICIs in UM showed limited results in terms of efficacy, whereas they demonstrated a favorable tolerability of these agents (Table 1).

Increasing evidence demonstrates that uveal melanoma cells employ escape mechanisms to elude the immune system [34]. The environment of the eye is an important immune-privileged site, with many immunosuppressive mechanisms that primary UM cells retain to gain immune-protection even when they leave the eye. The immune-modulatory microenvironment of the liver, the typical site of metastasis for UM, could further protect escaped UM cells from immune surveillance [35]. Furthermore, the low mutational burden is considered another relevant characteristic of uveal melanoma, which could justify the limited activity of immunotherapy [36].

Nevertheless, a minority of patients treated with immunotherapy obtain a clinical benefit [18].

In this review, we aim to describe the immunological features of UM in order to understand if it could still be a rationale for using immunotherapy in this disease.

## 2. Microenvironment of the Eye

The eye is considered a “privileged immunological site” [35] due to different mechanisms of immune protection.

The first is represented by the blood–ocular barrier: the tight junctions and the lack of lymphatic vessels in the cornea and uvea limit the circulation of immune cells [37].

The other mechanism involved is represented by soluble immunosuppressive factors in the aqueous humor [38]. They are: transforming growth factor (TGF)-β, α-melanocyte-stimulating hormone (α-MSH), calcitonin gene-related peptide (CGRP), vasoactive intestinal protein (VIP), and indoleamine 2,3 dioxygenase (IDO). TGF-β inhibits the activation of macrophages, T lymphocytes, and natural killer (NK) cells and enhances the tolerance of antigen-presenting cells (APCs) [34]. TGF-β is also required for CTLA-4 up-regulation on CD8^+^ T cells. CTLA-4 stimulation leads to T cell inactivation and generation of T regulatory (Treg) cells [39]. α-MSH reduces neutrophil activities and stimulates Tregs. α-MSH and CGRP downregulate the production of pro-inflammatory factors. VIP inhibits NK-cells mediated cytolysis. VIP and IDO inhibit T cell activation [34].

Moreover, the cornea, iris, and retina cells express immunosuppressive ligands on their surface, such as PD-L1 and FasL. PD-L1 suppresses proliferation and induces T-lymphocyte and neutrophil apoptosis when it recognizes its ligand, PD-1, on these cells. First apoptosis signal ligand (FasL, a member of the TNF family) promotes apoptosis of activated T cells [34]. Furthermore, complement regulatory proteins (CRPs) are capable of interrupting the complement cascade with the inhibition of complement-mediated cytolysis in the eye [40].

Corneal and retinal cells express MHC-Ib molecules (such as HLA-G and HLA-E); in this way, they are able to inhibit natural killer cell cytotoxic activity, binding the inhibitory receptors, such as CD94-NKG2. The interaction between MHC-I molecules and inhibitory receptors delivers ‘‘off signals’’ to NK cells, blocking their ability to kill target cells. On the other hand, the MHC class Ia molecules are down-regulated on corneal and retinal cells, reducing the susceptibility to T-cell mediated cytolysis [34].

When antigens enter the eye, a unique mechanism of immune privilege is involved: it is termed “anterior chamber-associated immune deviation” (ACAID), an immunomodulatory phenomenon involving the eye, but also the thymus, spleen, and sympathetic nervous system [41]. In mouse models an antigen injected into the anterior chamber of the eye is captured by ocular APCs. Then, these APCs migrate to the spleen and thymus, inducing immunomodulatory cells, such as Tregs, regulatory B cells, and NK T cells, which cause immune deviation. The sympathetic nervous system promotes the maintenance of a functional ACAID [42].

## 3. Microenvironment of the Liver

The liver is considered an immuno-modulatory organ whose microenvironment could promote tumor metastasis and growth [43]. Among the most relevant resident hepatic cells, the liver sinusoidal endothelial cells (LSECs) work as antigen-presenting cells and can cross-present antigens to T cells, perform receptor-mediated endocytosis, contribute to lymphocytes recruitment through chemokines production, and express PD-L1 in order to eliminate T cells [43,44]. LSECs also participate in immune tolerance by reducing MHC expression following the interaction with Kupffer cells (KCs) [35]. Molecules, such as interleukin-10 (IL-10) and prostaglandin E2 (PGE2) from KCs, promote immune tolerance of the liver reducing MHC expression on LSECs [35]. Hepatic stellate cells (HSCs) secrete IL-8, which is an angiogenic factor and favors proliferation of c-Met positive neoplastic cells. Tryptophan 2,3-dioxygenase (TDO) regulates tryptophan metabolism and induces differentiation of Treg cells suppressing T effectors and again promoting an immune suppressive microenvironment [45].

On the other hand, the liver can promote an immune response through different mechanisms. Hepatocytes themselves are able to express MHC molecules for immune responsivity. KCs are also APCs and can suppress metastases in the liver through the C-type lectin Dectin-2 [46]. Among liver circulating cells, T cells interact with APCs and different costimulatory molecules and, therefore, take part in the immune response or tolerance. Moreover, NK T cells secrete cytokines and chemokines, which contribute to immune responsivity or tolerance [47].

## 4. Immune Infiltrate in Uveal Melanoma

The crosstalk between tumor and microenvironment influences the inflammatory response: cancer cells interact with both the innate and the adaptive immune system and use immune cells for tumor survival and protection from immunological attacks. The main immune cells in uveal melanoma are the M2-type macrophages, which foster tumor growth through angiogenesis and immunosuppression [48,49].

**Figure 1:**
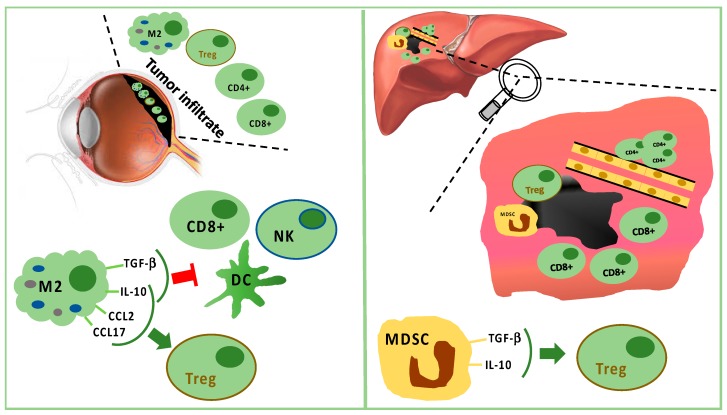
Tumor infiltrating immune cells in primary tumor (left panel) and in liver metastasis (right panel). In primary uveal melanoma (UM), CD8^+^ T cells and fewer CD4^+^ T cells are present, but M2 polarized macrophages are predominant. M2 macrophages stimulate Tregs through IL-10, CCL2, and CCL17. M2 macrophages suppress CD8^+^, NK, and DC secreting TGF-beta and IL-10. In liver metastasis, CD8^+^ cells surround but do not infiltrate the tumor mass, while CD4^+^ cells are present in perivascular aggregates. Furthermore, Tregs are stimulated by TGF-beta and IL-10 produced by MDSC.

Monocytes/macrophages of M2 lineage exceed T cells in tumor-infiltrating immune cells. They secrete anti-inflammatory cytokines (IL-10 and TGF-β), which inhibit dendritic cell activation, as well as T- and NK-cell functions [35,48] (Figure 1). Treg and UM cells trigger inflammation and M2-polarization through the production of factors, such as CCL22, CCL2, VEGF, M-CSF, TGF-β, IL-6, IL-10, CCL17, CCL22, PGE2, and endothelial monocyte-activating polypeptide (EMAP)-II [35,48,50]. Moreover, M2-macrophages can secrete soluble factors, which enhance the invasive capabilities of neoplastic cells, such as the melanoma inhibitory activity (MIA), a growth-regulatory protein, which inhibits the cellular adhesion to the extracellular matrix [51].

The analysis of uveal melanoma suspensions has revealed that the majority of tumor infiltrating lymphocytes (TILs) are CD8^+^ T cells with fewer CD4^+^ T cells [52]. Lagouros reported a limited number of Tregs in primary UM [53]. Nevertheless, the count of Tregs within primary tumor correlates with the development of systemic metastases. In addition, Tregs and cyclooxygenase-2 (COX-2) expression in primary tumors are associated with a poor prognosis [54]. Tregs are recruited by IL-10, chemokine (C–C motif) ligand (CCL) 17, and CCL22 produced by M2 macrophages [35]. 

Tumor-associated macrophages (TAMs) are a negative prognostic factor for UM [55]. Indeed, TAMs are associated with highly malignant tumors characterized by negative prognostic features, such as epithelioid cells, high microvascular density, intense pigmentation, and larger size [56]. TAMs seem to promote tumor growth through angiogenesis and metastatic spread [55,57].

In primary UM, the expression of HLA class I and II exerts a prognostic role [58]. It has been reported that, in primary tumors, down-regulation of HLA class I is a mechanism for evading CD8^+^ cell cytotoxicity. Therefore, tumors should be more sensitive to NK cells [59,60,61,62]. Nevertheless, only 50% of primary UM expresses MHC class I related chain (MIC) A and B, which are the ligands for NK cell receptor (NKG2D). In UM metastases, MIC A and B are absent. Consequently, the activity of NK cells in metastatic UM is limited. Vetter reported a single case of MIC expression induced after chemotherapy, suggesting a possible role for immunotherapy following cytotoxic therapy [59]. It has also been proved that UM cells are capable of producing the macrophage migration inhibitory factor (MIF), a cytokine that inhibits cytolytic activity of NK cells, contributing to tumor growth and metastatic spread [63,64,65]. The expression of FasL is a further mechanism in UM explaining the escape from NK cells [66]. Additionally, TILs and TAMs of UM produce IL-2 and IL-15, which bind receptors on tumor cells, promoting UM cell growth and reducing sensitivity to NK cell activity [67].

Moreover, the loss of the maturation marker CD-40 on APCs has been observed in primary UM. This lack does not allow correct lymphocyte T-mediated anti-tumor activity because of an inadequate functioning of APCs to induce T cell activation [68].

Overall, “inflammatory phenotype” has been proposed to define UM with infiltrating macrophages and lymphocytes in addition to a high expression of HLA class I and II molecules. It identifies tumors with a worse prognosis [48,49].

It is doubtless that the immune cells in the UM inflammatory phenotype do not stimulate an antitumor response. They contribute to angiogenesis, immunosuppression, tumor growth, and metastatic spread [69]. Moreover, comparing the circulating immune cells between primary and metastatic UM, a weaker immune surveillance has been spotted in case of metastases. Lower circulating CD3^−^CD56^dim^ NK cells, CD8^+^, and NK T cells, as well as an increase in Tregs and MSDCs have been detected during the metastatic phase. Furthermore, metastases are associated with increasing plasma levels of several miRNAs involved in immune regulation [70].

In the metastatic sites, CD4^+^ cells are present in perivascular aggregates, while CD8^+^ cells are scarce and mainly surround the liver metastases [71,72,73,74]. Tumor cells and resident hepatic cells recruit in metastatic sites two different types of myeloid-derived suppressor cells (MDSCs): monocytic MDSCs and polymorphonuclear MDSCs. Monocytic MDSCs, more frequent than polymorphonuclear MDSCs in metastases, promote neoplastic growth after their differentiation towards TAMs. MDSCs are able to induce Tregs releasing IL-10 and TGF-β [35,71] (Figure 1).

Nevertheless, Rothermel found that a subset of TILs associated with a lack of melanin pigmentation is more effective in antitumor response, similarly to TILs in cutaneous melanoma [75].

### 4.1. The Role of PD-1/PD-L1 Interaction in UM

UM cell lines are able to suppress T-cell activation by the expression of PD-L1 (B7-H1) [76,77]. However, in vivo a limited constitutive expression of PD-L1 on tumor cells and PD-1 on TILs has been proven in metastatic uveal melanoma compared with cutaneous melanoma [78]. As a matter of fact, in the metastatic sites, only 5% of uveal melanoma shows the expression of PD-L1, whereas PD-1 is expressed in about 51% of TILs [79]. Regarding primary UM, 40% of PD-L1 expression on tumor cells is reported in patients with metastatic disease [80].

PD-L1 expression indicates an active interaction between the tumor and the adaptive immune cells. PD-L1 expression is induced by IFN-γ produced by activated CD8^+^ cells. Consequently, PD-L1 on tumor cells depends on the presence of activated TILs PD-1^+^ [79]. The lower PD-L1 expression in UM is not due to a loss of function. Indeed, it is known that uveal melanoma cells do not lose the ability to upregulate PD-L1 in response to IFN-γ. In a preclinical model, PD-L1 is able to inhibit T cell proliferation through a lower secretion of IL2 [81].

Among PD-1/PD-L1 patterns, in UM samples the two more frequent patterns are PD-1^−^/PD-L1^−^ and PD-1^+^/PD-L1^−^, representing immunological tolerance, with, respectively, absence or functional suppression of TILs in the tumor microenvironment. Differently, in cutaneous melanoma the dominant subgroup is the PD-1^+^/PD-L1^+^, resulting in immune-competent and active TILs. This subgroup seems to be absent in UM. TILs with antitumor function are less frequent than regulatory TILs in UM, resulting in immune tolerance both in the primary site and metastases. [79]. PD-1/PD-L1 axis is probably not one of the most relevant mechanisms to avoid immune response in uveal melanoma. This hypothesis can explain the poor response to anti-PD-1 therapy.

### 4.2. IDO and Immune Escape

The enzyme indoleamine 2,3 dioxygenase (IDO) controls tryptophan degradation, influencing both the innate and the adaptive immune system for lymphocytes proliferation, activation, and survival. IDO is able to suppress T and NK cells, generate Tregs, and promote tumor angiogenesis.

In uveal melanoma IDO is not constitutively expressed either in primary or in metastatic cells. However, IFN-γ upregulates IDO mRNA and protein in UM cells, inducing the production of enzymatically and biologically active IDO [82,83]. We might conclude that the induction of IDO by IFN-γ is a defensive mechanism of uveal melanoma cells in response to the presence of T lymphocytes and NK cells. Upregulation of the IDO gene and protein expression can contribute to an immune-privileged microenvironment promoting immune escape. In advanced cutaneous melanoma, epacadostat, an IDO1 inhibitor, in addition to anti-PD-1 treatment, does not improve the clinical outcomes of anti-PD-1 therapy alone [84]. The effectiveness of IDO inhibitors in UM could be limited by the lack of a constitutive expression. Nevertheless, we cannot exclude a role of IDO inhibitors in selected patients or in special conditions inducing IDO expression (i.e., previous treatments).

## 5. Mutational Burden and Epitopes of UM

Uveal melanoma is a relatively simple genetic disease characterized by recurrent chromosomal losses and gains determining a low mutational rate. Monosomy 3, 1p loss, 1q gain, 6q loss, 6p gain, 8p loss, and 8q gain are the most frequent chromosomal abnormalities in uveal melanoma [85,86,87,88]. Monosomy of chromosome 3 has been associated with poor prognosis and metastatic behavior [89,90]. Chromosome 3 monosomy has been correlated with the inflammatory phenotype: analyzing 50 tissue samples of UM, an association between monosomy 3 and higher expression of TAMs and class I and II HLA expression was emphasized. It has been supposed that the inactivation of the peroxisome proliferator-activated receptor (PPARγ) located on chromosome 3 can increase factors that favor the inflammatory microenvironment [91]. BAP1 (located on 3p21.31–p21.2) is the most commonly mutated oncosuppressor (inactivating mutations detected in up to 47% of UM), which seems to be associated with high TAMs infiltration [92]. GNAQ/GNA11 is the most commonly mutated oncogene, associated with constitutive activation of the RAS-ERK pathway [93,94]. SF3B1 mutations are evident in approximately 15% of cases, associated with better prognosis [36].

The low mutational burden of UM and the consequent small number of epitopes available for immune response might explain the observed limited efficacy of immunotherapy, such as anti-CTLA-4 or anti-PD-1.

Recently, it has been described that a defect of MBD4, a transcriptional factor of gene promoters, is associated with a hyper-mutated CpG>TpG pattern, which generates multiple sub-clones of the primary UM with more heterogeneous metastases and high mutational burden. A patient with this MBD4-related hyper-mutator phenotype exhibited a remarkable response to immune checkpoint inhibitor [95]. This evidence implies that specific and selected subgroups of UM could benefit from immunotherapy.

UM shows high expression of glycoprotein 100 (gp100), melanoma-associated antigen (MAGE), melanoma antigen recognized by T cells (MART-1), and tyrosinase-related protein-1 (TRP-1), which are cancer antigens known to be immunogenic [96,97,98]. They represent future perspectives for UM therapy. For example, IMCgp100 (directed against gp100) is currently being tested as a new approach to UM immune-based therapy. Dendritic cell vaccinations directed against antigens that are highly expressed in UM, such as gp100, represent promising approaches for future therapies, since autologous dendritic cells loaded with antigens detected in UM were able to induce immune responses in 11 treated patients. Larger cohorts of patients are required to confirm this hypothesis [99].

## 6. Conclusions

As patients with UM were excluded by clinical trials of ICIs in melanoma, data with ICIs in UM are mainly based on retrospective studies (including from five to 100 patients) and on observational or phase II trials enrolling from 11 to 53 patients in different clinical settings (Table 1). Median PFS reported ranges were from 2.3 to 3.0 months and median OS from 5.2 to 14 months. It is worth underlining that the reported response rates ranged from 0 to 30% [100]. The responding patients achieved a remarkable survival [18].

Some features of uveal melanoma, such as the privileged site of the eye, the immune suppressive microenviroment of the liver, and the low mutational burden associated with several mechanisms of immune escape can explain the poor results obtained with anti-PD-1 and anti-CTLA-4 agents in metastatic uveal melanoma. 

However, the immune system seems to play a remarkable role also in this disease. We cannot exclude that immunological treatments based on mechanisms that differ from PD-1 and CTLA-4 could yield better results. The trend towards better survival outcomes in patients with some types of autoimmune disease in UM strengthens the importance of the immune system in this malignancy, suggesting a possible role for immunotherapy in the treatment [101].

Future therapeutic options in mUM include treatments such as adoptive transfer of autologous TILs [102,103], requiring a deeper identification and characterization of more reactive TILs in UM and a better elucidation of suppressive pathways. Dendritic cell vaccination loaded with specific UM antigens could represent a future strategy for treating this disease [98]. Indeed, loading dendritic cells with specific tumor antigens could increase the capability of CD8^+^ T cells to infiltrate tumor masses [104]. Therefore, antigens that are typically expressed by UM, such as gp100, could become a therapeutic target to enhance immunoreactivity. 

Further knowledge on the immunological backbone of UM could allow the selection of patients who may benefit more from immunotherapy. Additional studies are needed to unravel the suppressive mechanisms of UM and identify new targets to enhance anti-tumor immunoreactivity. 

## Figures and Tables

**Table 1:** Clinical studies with immune checkpoint inhibitors (ICIs) in uveal melanoma.

Authors	Treatment	Type of Study	No. of Enrolled Patients	Year
Zimmer et al. [19]	Ipilimumab	Phase II trial. Pre-treated and naïve patients.	53	2015
Maio et al. [17]	Ipilimumab	Retrospective analysis. Pre-treated patients.	82	2013
Kelderman et al. [20]	Ipilimumab	Retrospective analysis. Pre-treated patients.	22	2013
Luke et al. [15]	Ipilimumab	Retrospective, multi-center analysis. Pre-treated and naïve patients.	39	2013
Piulats Rodriguez et al. [21]	Ipilimumab	Phase II trial. Naïve patients	32	2014
Danielli et al. [10]	Ipilimumab	Retrospective analysis. Pre-treated patients.	13	2012
Khattak et al. [13]	Ipilimumab	Retrospective analysis, single center analysis. Pre-treated patients.	5	2016
Deo [22]	Ipilimumab	Retrospective, single center analysis. Pre-treated patients.	24	2014
Shaw et al. [23]	Ipilimumab	EAP.	18	2012
Joshua et al. [11]	Tremelimumab	Phase II trial. Naïve patients.	11	2015
Algazi et al. [9]	Pembrolizumab, Nivolumab, Atezolizumab	Retrospective, multi-center analysis. Pre-treated and naïve patients.	56	2016
Mignard et al [16]	Pembrolizumab, Nivolumab, Ipilimumab	Retrospective, multi-center analysis.	100	2018
Bender et al. [27]	Pembrolizumab, Nivolumab	Retrospective, multi-center analysis. Pre-treated patients.	15	2017
Heppt et al. [24]	Pembrolizumab, Nivolumab, Ipilimumab	Retrospective, multi-center analysis. Pre-treated and naïve patients.	96	2017
Piperno-Neumann et al. [25]	Pembrolizumab, Nivolumab	Retrospective, single center analysis. Naïve patients.	21	2016
Karydis et al. [12]	Pembrolizumab	Retrospective analysis. Pre-treated patients.	25	2016
Rossi et al. [18]	Pembrolizumab	Prospective. Naïve patients.	17	2019
Kottschade et al. [14]	Pembrolizumab	Retrospective, single-center analysis. Pre-treated patients.	8	2016
Van der Kooij et al. [26]	Pembrolizumab	Prospective. Pre-treated and naïve patients.	17	2017
Schadendorf et al. [27]	Nivolumab	Phase II. Pre-treated patients.	75	2017
Jung et al. [28]	Ipilimumab	Named patient use. Pre-treated patients.	10	2017
Shoushtari et al. [29]	Nivolumab, Ipilimumab	Expanded access program.	6	2016

EAP: Expand Access Program.
